# The Novel Role of HtrA1 in Gingivitis, Chronic and Aggressive Periodontitis

**DOI:** 10.1371/journal.pone.0096978

**Published:** 2014-06-30

**Authors:** Teresa Lorenzi, Elena Annabel Niţulescu, Antonio Zizzi, Maria Lorenzi, Francesca Paolinelli, Simone Domenico Aspriello, Monica Baniţă, Ştefania Crăiţoiu, Gaia Goteri, Giorgio Barbatelli, Tommaso Lombardi, Roberto Di Felice, Daniela Marzioni, Corrado Rubini, Mario Castellucci

**Affiliations:** 1 Department of Experimental and Clinical Medicine, Università Politecnica delle Marche, Ancona, Italy; 2 Department of Histology, University of Medicine and Pharmacy of Craiova, Craiova, Romania; 3 Pathological Anatomy, Department of Medical Sciences and Public Health, Università Politecnica delle Marche, United Hospitals, Ancona, Italy; 4 Department of Clinical Specialistic and Dental Sciences, Periodontology, Università Politecnica delle Marche, Ancona, Italy; 5 Laboratory of Oral and Maxillofacial Pathology, Division of Stomatology and Oral Surgery, University of Geneva, Geneva, Switzerland; 6 Private Dental Practice, San Benedetto del Tronto, Ascoli Piceno, Italy; University of Oklahoma Health Sciences Center, United States of America

## Abstract

Proteolytic tissue degradation is a typical phenomenon in inflammatory periodontal diseases. HtrA1 (High temperature requirement A 1) has a serine protease activity and is able to degrade fibronectin whose fragments induce the expression and secretion of several matrix metalloproteinases (MMPs). The aim of this study was to investigate for the first time if HtrA1 has a role in gingivitis and in generalized forms of chronic and aggressive periodontitis. Expression of HtrA1 was investigated in 16 clinically healthy gingiva, 16 gingivitis, 14 generalized chronic periodontitis and 10 generalized aggressive periodontitis by immunohistochemistry and real-time PCR. Statistical comparisons were performed by the Kruskall-Wallis test. Significantly higher levels of HtrA1 mRNA and protein expression were observed in pathological respect to healthy tissues. In particular, we detected an increase of plasma cell HtrA1 immunostaining from gingivitis to chronic and aggressive periodontitis, with the higher intensity in aggressive disease. In addition, we observed the presence of HtrA1 in normal and pathological epithelium, with an increased expression, particularly in its superficial layer, associated with increasingly severe forms of periodontal disease. We can affirm that HtrA1 expression in plasma cells could be correlated with the destruction of pathological periodontal tissue, probably due to its ability to trigger the overproduction of MMPs and to increase the inflammatory mediators TNF-α and IL-1β by inhibition of TGF-β. Moreover, epithelial HtrA1 immunostaining suggests a participation of the molecule in the host inflammatory immune responses necessary for the control of periodontal infection.

## Introduction

Periodontal diseases are characterized by an immune response to antigens in bacterial plaque as well as by alterations in connective tissue and morphological changes in the epithelium. This response is always clinically evidenced as gingivitis or periodontitis [1], [2]. Gingivitis, a reversible inflammatory lesion, is the initial stage of gingival disease and the easiest to treat [3]. On the contrary, the periodontitis are characterized by the chronic activation of the immune system, alterations in connective tissue metabolism, production of cytokines and proteinases as well as the direct destruction of hard and soft tissue structures which support teeth by bacterial enzymes and virulence factors [3]. Among all the forms of periodontitis, chronic and aggressive periodontitis have received considerable attention due to their peculiar clinical presentation [4].

Interestingly, matrix metalloproteinases (MMPs) produced by immunoregulatory cells and fibroblasts play a destructive role in the inflammatory periodontal lesion progression [5]. It has been proposed that the expression and secretion of several MMPs are enhanced by fragments coming from the degradation of fibronectin [6], [7]. This extracellular matrix glycoprotein is a natural substrate of HtrA1 (High temperature requirement A 1), a member of the family of HtrA proteins with serine protease activity [8]. HtrA1 is a secreted multidomain protein and it is characterized by the presence of a highly conserved trypsin-like serine protease domain and at least one protein interaction PDZ (PSD-95 (Postsynaptic density protein of Mr 95 kDa), Dlg (Drosophila Discs-Large protein) and ZO-1 (Zonula occludens protein 1)) domain [9] It also contains insulin-like growth factor-binding protein/follistatin/Mac25-like domain and a Kazal-type serine protease inhibitor motif at its N-terminus [9]. Serine protease HtrA1 is implicated in physiologic processes, such as the inhibition of TGF-β signalling [10], and in the pathogenesis of various diseases, including osteoarthritis, Alzheimer’s disease and preeclampsia [7], [8], [11]. Since HtrA1 plays a role in extracellular matrix degradation acting directly by proteolytic cleavage of matrix protein components [12], or indirectly through HtrA1 ability to stimulate the overproduction of MMPs [7], we hypothesized an important role for HtrA1 in the onset of tissue destruction characterizing inflammatory periodontal diseases.

The aim of this study was to examine, for the first time, the profile of HtrA1 protein and mRNA expression by immunohistochemistry and real-time PCR, respectively, in gingivitis and in generalized forms of chronic and aggressive periodontitis, compared to clinically healthy gingiva. Our findings highlight a correlation of plasma cell HtrA1 expression with the occurrence of periodontal diseases and a possible contribution of epithelial HtrA1 to the control of periodontal infection.

## Materials and Methods

### Ethics statement

The experimental protocol was approved by the Ethics Committee of Università Politecnica delle Marche for human subjects and the study was conducted in accordance with the Helsinki Declaration of 1975, as revised in 2000. Written informed consent was granted from patients and parents on the behalf of the minors/children participants prior to inclusion in this study and recorded on file.

### Individuals and Gingival Tissue Samples

The clinical and periodontal parameters of subjects were assessed by a single examiner (S.D.A.), as previously described [13]. A University of North Carolina (UNC)-15 calibrated periodontal probe (15 mm, probe tip diameter  = 0.5 mm) (Hu Friedy, Chicago, IL, USA) was used for all the measurements.

Gingival tissue samples from 16 clinically healthy gingiva and 16 gingivitis (Table 1) were obtained from premolars of individuals not affected by periodontitis and showing no supporting tissue destruction, collected from sites requiring dental extraction for orthodontic treatment. Furthermore, 24 gingival specimens were collected from premolars of adults with periodontitis (14 generalized chronic periodontitis and 10 generalized aggressive periodontitis) (Table 1) during routine periodontal flap surgery after the initial phase of periodontal therapy, consisting of scaling and root planing. The clinical diagnosis of generalized chronic periodontitis was based on the presence of up to 30% of measured sites with clinical attachment loss (CAL) >5 mm [14]. The clinical diagnosis of generalized aggressive periodontitis was made in patients, aged between 18 and 40 years, showing an attachment loss greater than 6 mm affecting at least three permanent teeth other than the first molars and incisors [14]. In all the cases the histological framework was coherent with the clinical diagnosis.

**Table 1:** Clinical profile of gingival biopsy sites.

Individuals	N	Age (years)	Male	Gingival Index(mean ± SD)	Probing depth(mm) (mean ± SD)	Sulcus BleedingIndex (mean ± SD)
		mean ± SD* range				
Healthy	16	23.87±5.53 17–35	8	0	2.50±0.43	0
Gingivitis	16	37±9.39 23–51	8	1.87±0.33	3.05±0.60	2.65±0.48
ChronicPeriodontitis	14	47.25±5.52 42–57	6	2±0.70	6.87±0.78	4.12±0.60
AggressivePeriodontitis	10	25±4.55 20–35	5	2.12±0.78	7.37±1.11	4.62±0.48

*SD: Standard Deviation.

The periodontal parameters (gingival index, probing depth and sulcus bleeding index) [13], were assessed at six different sites around each tooth (mesio-buccal, mid-buccal, disto-buccal, mesio-lingual, mid-lingual and disto-lingual). All the teeth enrolled in the study had homogeneous clinical parameters on all the six sites analyzed. The clinical status related to gingival biopsy sites is shown in Table 1.

None of the study participants: (i) had systemic diseases (endocrine disorders, immunodepression, diabetes), (ii) had used antibiotics, corticosteroids or non-steroidal anti-inflammatory drugs within the preceding 6 months, (iii) smoked or (iv) had used calcium channel-blockers, cyclosporine A, phenytoin, or any other drugs associated with gingival hyperplasia. Moreover, subjects who have undergone periodontal treatment within the previous 2 years were discarded.

From each subject, we obtained gingival tissue biopsies (n = 2) containing oral epithelium and subepithelial soft tissues, with a cubic shape of approximately 3 mm in length. In addition, first trimester placental tissue was used as positive control for real-time PCR [15], and for immunohistochemistry techniques [16], because it expresses both HtrA1 mRNA and protein.

For immunohistochemical analysis, specimens were fixed for 24 hours in 4% neutral buffered formalin at 4°C, and then embedded in paraffin. For molecular (real time-PCR) analysis, tissues were immediately frozen in liquid nitrogen and stored at –80°C.

### Immunohistochemistry

3-µm paraffin-embedded tissue sections were deparaffinised and incubated for 60 min with 3% hydrogen peroxide in deionised water to inhibit endogenous peroxidase activity. Sections were washed in 50 mM Tris/HCl, pH 7.6 and pre-treated for 10 min at 98°C in 10 mM sodium citrate, pH 6.0. After blocking non-specific background for 1 hr at room temperature (RT) with Tris/HCl-6% non fat dry milk (Bio-Rad Laboratories, Milan, Italy), sections were incubated overnight at 4°C with rabbit polyclonal anti-human HtrA1 antibody (Abcam, Cambridge, UK), diluted 1∶100 in Tris/HCl-3% non-fat dry milk. After washing in Tris/HCl, the sections were incubated for 1 hr at RT with goat anti-rabbit biotinylated antibody (Vector Laboratories, Burlingame, CA), diluted 1∶200 in Tris/HCl-3% non fat dry milk. The peroxidase ABC method (Vector Laboratories) was applied for 1 hr at RT using 3′, 3′ diaminobenzidine hydrochloride (Sigma Chemical Co, St Louis, MO, USA) as the chromogen. Sections were counterstained in Mayer’s haematoxylin, dehydrated and mounted with Eukitt solution (Kindler GmbH and Co., Freiburg, Germany). Negative controls were performed for gingival and placental tissues by omitting the primary or the secondary antibody. An isotype control antibody (Rabbit IgG, cat. n° I-100, Vector Laboratories, diluted 1∶150) was used as a further negative control. The negative controls confirmed the specificity of the immunolabelling obtained with the primary antibody.

Two investigators evaluated simultaneously the cytoplasmic HtrA1 immunoreactivity in the more representative fields using images of the histological sections captured with a digital system. For this purpose, each field with area of 0.22 mm^2^ was captured with a camera coupled to the light microscope and to a computer for digitalization of the image (Nikon Spa, Firenze, Italy). HtrA1 protein staining was classified with a five-point scale and was assigned a number value to each point: negative (– = 0), slight (+/− = 1), faint (+ = 2), moderate (++ = 3) and strong (+++ = 4). The evaluation of HtrA1 immunoreactivity provided by the two examiners was similar.

### Preparation of cDNA for Q-PCR

Total RNA was extracted from frozen tissues (10 mg) using the Total RNA purification kit (Norgen, Biotek Corp., Thorold, Ontario, Canada), and then cleaned up and concentrated using the CleanAll RNA/DNA Clean-Up and Concentration Kit (Norgen), according to the manufacturer’s instructions. The quality (A_260_/A_280_) and quantity (A_260_) of extracted RNA were tested by a NanoDrop ND-1000 UV-Vis Spectrophotometer (Celbio, Milan, Italy). The integrity of the isolated RNA was checked by 1.5% denaturating agarose gel. 1 µg of RNA was reverse transcribed by the high-capacity cDNA RT kit (Applied Biosystems, Foster City, CA) using random primers.

### Real-time PCR (Q-PCR) analysis

The sequences of the Q-PCR primers targeting HtrA1 gene are reported in Table 2. The reference gene SDHA (Succinate Dehydrogenase Complex Subunit A) was used as housekeeping gene for data normalization in order to correct for variations in RNA quality and quantity.

**Table 2:** Characteristics of the primers used for SYBR green Q-PCR assays.

Target gene	Primer*	Primer sequence (5′-3′)	Tm(°C)†	%GC	Ampliconlength (bp)	Accession no.
HumanHtrA1	hHTRA1_FhHTRA1_R	GGAAGATGGACTGATCGTGA CATAGTTGATGATGGCGTCG	57.3 57.3	50 50	347	NM_002775
HumanSDHA	hSDHA_FhSDHA_R	AGCATCGAAGAGTCATGCAG TCAATCCGCACCTTGTAGTC	57.3 57.3	50 50	398	NM_004168

*Letters F and R after the primer name indicate forward and reverse orientation, respectively.

† Theoretical melting temperature (*Tm*) calculated using the MWG Oligo Property Scan (MOPS).

Real-time PCR was performed in a reaction mixture containing 10 µl of 2X iQ SYBR Green Supermix (Bio-Rad Laboratories), 0.1 µM of each primer, 0, 3 ng of sample template and RNase-free sterile water to a final volume of 20 µl. Amplification was performed using the iQ5 Multicolor Real-Time PCR Detection System (Bio-Rad Laboratories) using the following program: (i) an initial denaturing step at 95°C for 15 min.; (ii) 45 cycles, with 1 cycle consisting of denaturation at 95°C for 10 s, annealing at 60°C for 30 s and extension at 72°C for 30 s. Q-PCR assays with CT values over 40 were considered negative. For each PCR run, a negative (no-template) control and a minus-reverse transcriptase (“-RT”) control were used to test for false-positive results or DNA contamination, respectively. The absence of non-specific products or primer dimers was confirmed by observation of a single melting peak in a melting curve analysis. For each Q-PCR, the genes were run in duplicate and all samples were tested in three separate experiments. In addition, the standard curve for each gene was constructed using serial dilutions of the cDNA obtained from the first trimester placenta (positive control).

Since PCR efficiencies were found to be close to 100%, the 2^−ΔΔCt^ (Livak) method was applied to compare data from gingivitis and chronic/aggressive periodontitis to the healthy gingival tissues (reference group). The results were obtained as “fold changes” in relative gene expression of tissues affected by the periodontal diseases respect to the reference ones.

### Statistical analysis

A Kruskall-Wallis test was performed for evaluating RT-PCR and immunohistochemical differences between groups. A level of probability of 0.05 was used to assess the statistical significance. Statistical analyses were performed using the SPSS 16 package (SPSS Inc., Chicago, IL, USA). Data were expressed as median and 1^st^–3^rd^ quartiles.

## Results

### Histopathological features

**Figure 1:**
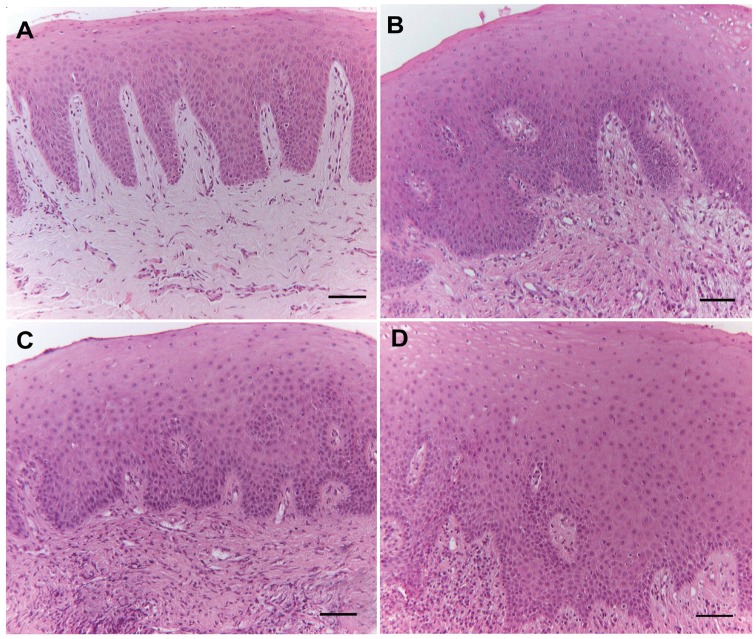
Haematoxylin and Eosin staining of periodontal tissues. **A)** healthy gingiva; **B)** gingivitis tissue; **C)** chronic periodontitis tissue; **D)** aggressive periodontitis tissue. Original magnification: 200x; scale bar: 50 µm.

Healthy gingival mucosa showed parakeratinized stratified squamous epithelium (Fig. 1A) and papillomatosis with rete pegs and connective tissue papillae. The connective tissue (or corium) was fibrous with some fibroblasts and low inflammatory cell infiltration, mainly in the marginal gingival zone (Fig. 1A).

Gingivitis specimens showed connective tissue with high vascularization and variable inflammatory cell infiltrates (Fig. 1B). At higher magnification we observed some plasma cells, indicating focal stimulation of the humoral immune system, but macrophages, neutrophils and lymphocytes were also present (data not shown). Epithelium was acanthotic and parakeratinized (Fig. 1B).

Chronic (Fig. 1C) or aggressive (Fig. 1D) periodontitis exhibited an epithelium lining the pockets that varies in thickness and sometimes was hyperplastic and acanthotic. In the underlying corium, a high vascularization and a high-grade inflammatory infiltrate were present, consisting mainly of lymphocytes, macrophages and plasma cells in varying proportions (data not shown).

### HtrA1 immunohistochemistry

**Figure 2:**
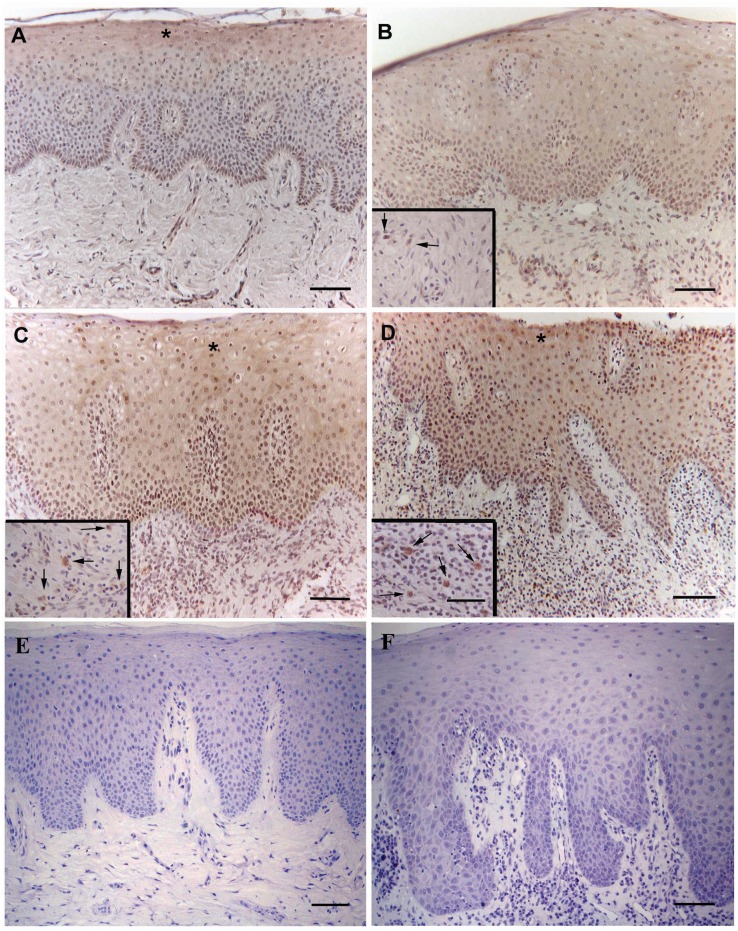
Expression of HtrA1, assessed by immunohistochemistry, in periodontal tissues. **A)** healthy gingiva; **B)** gingivitis tissue; **C)** chronic periodontitis tissue; **D)** aggressive periodontitis tissue. Negative control for healthy and aggressive periodontitis tissue is shown in panels **E)** and **F)**, respectively. Insets show the plasma cells positive to HtrA1 (arrows). The asterisk indicate the layer with the strongest HtrA1 immunostaining. Original magnification: 200x; scale bar: 50 µm. Original magnification of insets: 400x; scale bar: 25 µm.

**Figure 3:**
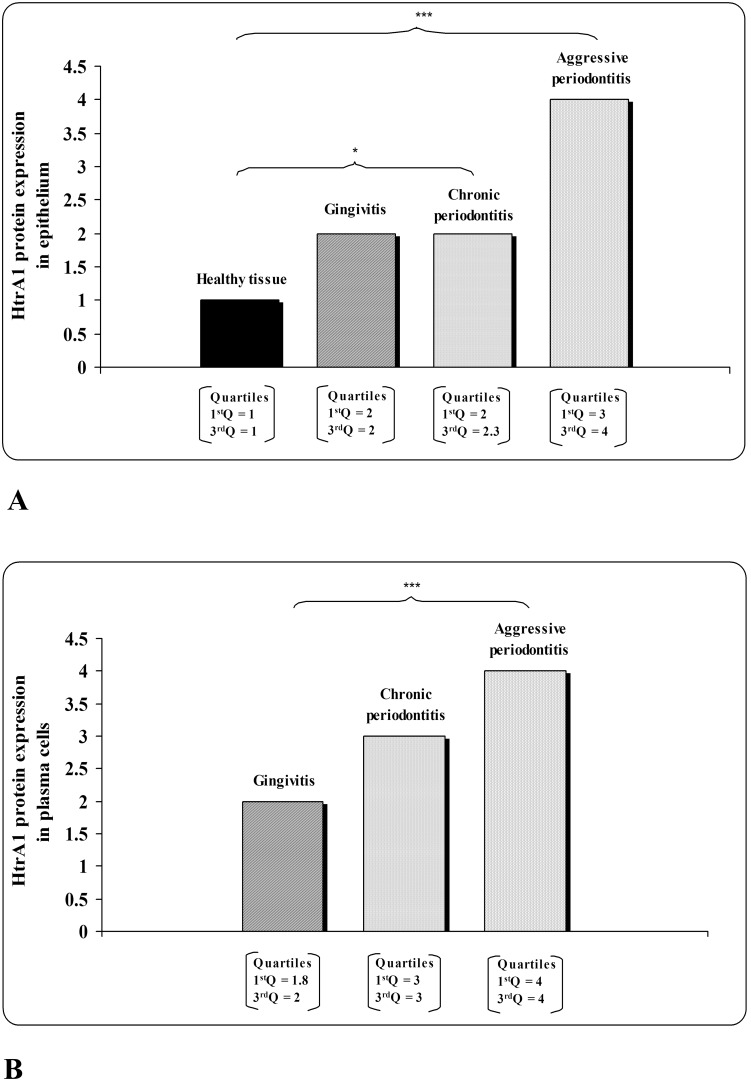
HtrA1 protein expression in A) epithelium and in B) plasma cells of studied groups. HtrA1 expression was classified with a five-point scale and was assigned a number value to each point: negative (neg = 0), slight (+/− = 1), faint (+ = 2), moderate (++ = 3) and strong (+++ = 4). p<0.05 (*); p<0.001 (***).

HtrA1 showed a *cytoplasmic* staining. First trimester placental tissue (positive control) was positive for HtrA1 (data not shown). Healthy gingival tissues (Fig. 2A) revealed a gradual increase in HtrA1 immunoreactivity from the basal to the superficial layer of epithelium (Table 3). In gingivitis sections (Fig. 2B), HtrA1 positivity resulted uniformly distributed in all layers (Table 3). In chronic (Fig. 2C) and aggressive (Fig. 2D) periodontitis we observed HtrA1 immunostaining in the whole thickness of the epithelium, but with a stronger intensity in the superficial layer (Table 3). In addition, aggressive periodontitis showed a more evident HtrA1 positivity in all layers than chronic periodontitis (Table 3). Our results demonstrated an increase of HtrA1 immunostaining in epithelium, particularly in its superficial layer, associated with increasingly severe forms of pathology (Table 3, Fig. 2). In addition, parakeratotic layer showed a negative HtrA1 reactivity in all studied groups (Fig. 2). Thus, the whole epithelium showed a significant increase of HtrA1 expression in chronic (p = 0.037) and aggressive (p<0.001) periodontitis compared to healthy subjects (Fig. 3A). In the corium of inflamed gingival tissue, HtrA1 was mainly expressed in plasma cells (Table 3, Figs. 2-insets, 3B). Gingivitis samples contained a few faintly (Table 3, Figs. 2B, 3B) immunopositive plasma cells. In chronic and aggressive periodontitis plasma cells reactive to HtrA1 were more represented and strongly stained than in gingivitis (Fig. 2C, 2D), with an increasing intensity of the immunostaining from chronic (Table 3, Figs. 2C, 3B) to aggressive (p<0.001) (Table 3, Figs. 2D, 3B) periodontitis.

**Table 3:** Immunohistochemical staining for HtrA1 in studied groups.

	HEALTHY	GINGIVITIS	CHRONIC PERIODONTITIS	AGGRESSIVE PERIODONTITIS
**EPITHELIUM**				
Superficial	+	+	++	+++
Intermediate	+/−	+	+	++
Parabasal	−	+	+	++
Basal	+/−	+	+	++
**DERMIS**				
Collagen fibers	−	−	−	−
Fibroblasts	−	+	−	−
Plasma cells	/	+	++	+++
**VESSELS**	+	+	+	+

Fibroblasts and collagen fibers resulted negative for HtrA1 in all cases, except in gingivitis where fibroblasts were faintly positive (Table 3, Fig. 2B). HtrA1 immunostaining intensity in dermal vessels was weakly positive in all groups (Table 3, Fig. 2).

### Expression of HtrA1 mRNA in healthy and pathological gingival tissues

**Figure 4:**
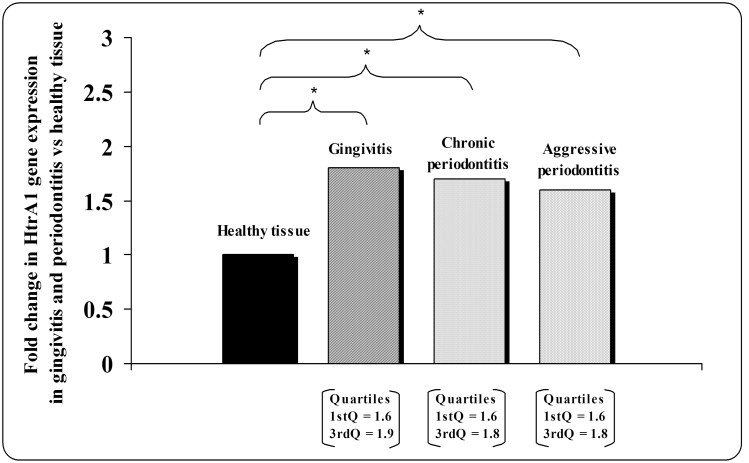
Quantitative real-time PCR of HtrA1 in periodontal tissues. Data are expressed as “fold changes” in relative gene expression of HtrA1 in periodontal diseases respect to the normal tissue. HtrA1 mRNA expression profile shows a statistically significant upregulation of its transcript in the analyzed periodontal diseases respect to healthy tissue. p<0.05 (*); p<0.01 (**).

Results of quantitative real-time PCR indicate that HtrA1 transcript was present in first trimester placental tissue (positive control) (data not shown) and in gingivitis at levels that are ∼2 fold higher than in healthy gingiva. This difference resulted statistically significant (p<0.001) (Fig. 4). Real-time PCR analysis of HtrA1 in chronic and aggressive periodontitis showed higher levels of mRNA expression than healthy tissues. Also in these cases, significant differences were detected (p = 0.004 and p = 0.009 for chronic and aggressive periodontitis, respectively) (Fig. 4). The differences of mRNA expression among the analyzed pathologies were not significant.

## Discussion

Periodontal diseases are infectious inflammatory pathologies characterized by the destruction of the tooth-supporting structures [17]. Besides the innate immune response, the mobilization of adaptive immunity mechanisms induced by periodontal bacteria are the leading causes of inflammation [18]. Indeed, we confirm an increase in the number of immunoglobulin-producing plasma cells in chronic and aggressive periodontitis compared to gingivitis specimens [19]. Interestingly, we found a greater amount of plasma cells positive to HtrA1 in the periodontitis lesions respect to gingivitis. In addition, we detected an increase of plasma cell HtrA1 immunostaining from gingivitis to chronic and aggressive periodontitis, with the higher intensity in aggressive disease.

It is known that HtrA1 is able to digest fibronectin [8], and that the accumulation of fibronectin fragments instigates the expression and secretion of several MMPs [7]. MMPs, a family of zinc- and calcium-dependent proteases, have been often associated with the remodelling of periodontal tissues [20], because, together with tissue inhibitors of metalloproteinases (TIMPs), are supposed to control the extracellular matrix physiological turnover [21]. The *plasma cell expression* of HtrA1 *protein* in periodontal diseases, high in chronic but stronger in aggressive periodontitis, could trigger the overproduction of matrix metalloproteinases, causing an increase in the *MMP* **/** *TIMP* **-** *ratio* in periodontal tissues with the resulting tissue destruction. Moreover, it is known that HtrA1 functions as an inhibitor of transforming growth factor (TGF)-β signalling [10], and seems to be able to bind and inhibit signalling of a wide range of TGF-β family proteins [10]. TGF-β is a pleiotropic cytokine that down-regulates the transcription of pro-inflammatory molecules, such as *Tumor Necrosis Factor* **-**α (TNF-α), and MMPs [22], [23]. TNF-α plays a central role in inflammatory reaction in periodontal tissues. In particular, TNF-α up-regulates other classic pro-inflammatory innate immunity cytokines production, such as Interleukin-1β (IL-1β) [24]. We suggest that the strong HtrA1 positivity observed in plasma cells of tissues from patients affected by chronic and aggressive periodontitis could reduce the inhibitory effects of TGF-β, allowing the increase of inflammatory mediators (TNF-α; IL-1β) promoting disease progression. The lack of HtrA1 immunoreactivity in the pathological connective tissue rules out the involvement of the serine protease in the direct tissue destruction by proteolytic cleavage of matrix protein components. Therefore, the present study supports the hypothesis that HtrA1 protein expressed in plasma cells can indirectly take part in periodontal lesions, contributing to pathological tissue remodelling in periodontal diseases.

An essential feature found in chronic and aggressive periodontitis is the strong HtrA1 positivity in the epithelium, higher than in the epithelial layers of healthy and gingivitis specimens. Recent studies have demonstrated that TNF-α may also play important functions in the control of bacterial levels in the periodontal environment [25]. In addition, mouse models point to relevant role for TNF-α in the control of periodontal infection [26]. The epithelial immunolocalization of HtrA1 and its strong positivity, particularly in the upper part of the epithelium, suggest us that HtrA1 may have a role in controlling the environment outside the tissue. In particular, HtrA1 probably increases the transcription of TNF-α by inhibition of TGF-β signalling, thus acting as one factor of the immune protection necessary to counteract the growth, the invasion and the toxin production of bacteria organized as a dental biofilm in the deepened periodontal pockets [27]. The increase of HtrA1 immunostaining in the epithelial superficial layer associated with increasingly severe forms of periodontitis appears to confirm the correlation between HtrA1 expression and the altered periodontal environment.

Gingivitis, chronic and aggressive periodontitis showed significantly higher levels of HtrA1 mRNA and protein expression respect to healthy tissues. This behaviour provides the demonstration that HtrA1 expression in periodontal pathologies is genetically determined. HtrA1 protein expression in aggressive periodontitis was higher than gingivitis and chronic periodontitis, while no differences were found in HtrA1 mRNA levels between the three pathologies. This may show that HtrA1 gene expression is not a marker for a particular type of periodontitis but more probably for periodontal inflammation in general. The discrepancy between HtrA1 protein and gene expression is probably due to a possible regulation of HtrA1 mRNA translation by G-quadruplexes, structural elements formed in the 5′-UTRs of many genes recognized to influence the translation [28]. Another explanation could be provided by the 26S proteasome-mediated proteolysis degradation of proteins, such as HtrA1, involved in biological processes as control of cell proliferation and differentiation and programmed cell death (apoptosis) [29], [30]. Since deregulation of proteasome function is known to occur in various human diseases [30], HtrA1 proteolysis could be different among the analyzed periodontal pathologies.
